# Editorial: Whiplash-associated disorder—advances in pathophysiology, patient assessment and clinical management

**DOI:** 10.3389/fpain.2022.1071810

**Published:** 2022-11-10

**Authors:** Rutger M. J. de Zoete, Iris Coppieters, Scott F. Farrell

**Affiliations:** ^1^ School of Allied Health Science and Practice, The University of Adelaide, Adelaide, SA, Australia; ^2^ Pain in Motion Research Group (PAIN), Department of Physiotherapy, Human Physiology and Anatomy, Faculty of Physical Education & Physiotherapy, Vrije Universiteit Brussel, Brussels, Belgium; ^3^ Laboratory for Brain-Gut Axis Studies (LaBGAS), Translational Research Centre for Gastrointestinal Disorders TARGID, Department of Chronic Diseases and Metabolism (CHROMETA), KU Leuven, Leuven, Belgium; ^4^ RECOVER Injury Research Centre, The University of Queensland, Herston, QLD, Australia; ^5^ NHMRC Centre of Research Excellence: Better Health Outcomes for Compensable Injury, The University of Queensland, Herston, QLD, Australia; ^6^ Tess Cramond Pain & Research Centre, Royal Brisbane & Women’s Hospital, Herston, QLD, Australia

**Keywords:** neck pain, chronic pain, whiplash associated disorder, whiplash injuries, physical rehabilitation

**Editorial on the Research Topic** Whiplash-associated disorder—advances in pathophysiology, patient assessment and clinical management

“Whiplash injury’ refers to an acceleration-deceleration mechanism of injury to the cervical spine. Neck pain arising from such an injury—termed whiplash-associated disorder (WAD)—is the most common injury sustained in a motor vehicle crash (MVC) (1) and a substantial component of MVC compensation claims (2). In the Western world, around 300/100,000 people sustain a whiplash injury each year (3).

After a whiplash injury, approximately 50% of patients develop chronic WAD, with around 16% reporting ongoing severe pain-related disability (4). Despite decades of research, current guideline-based treatments such as education, advice and exercise, typically demonstrate modest effects (5–9). This may reflect our incomplete understanding of WAD's complex and heterogenous mechanisms, which span contributions from musculoskeletal (10, 11), neurological (12–19), inflammatory (20, 21) and psychological (22) factors, often within a “compensable injury” social context (4). In this special Research Topic *Whiplash-associated disorder—Advances in pathophysiology, patient assessment and clinical management*, we sought to bring together a series of papers that provide new insight into mechanisms underpinning WAD, considerations in clinical assessment, as well as approaches to patient management.

Post-traumatic stress symptoms (PTSS) and sensory hypersensitivity are established predictors of poor outcome after whiplash injury (23, 24) and likely contribute to mechanisms underlying patient symptoms. Andersen et al. build on this prior data with a large cohort study (*N* = 740) exploring PTSS, pain and quantitative sensory testing (pressure algometry) across a 12-month period following a whiplash injury. Those patients with clinical PTSS had sensory hypersensitivity across all time points as well as greater pain intensity and more widespread pain—demonstrating a clear link between PTSS, sensory hypersensitivity and clinical outcomes. These data elegantly reflect the biopsychosocial dimensions of WAD and clinical importance of PTSS, especially in the context of a recent clinical trial targeting PTSS after whiplash injury, with clinically meaningful effects on neck pain-related disability at 6 weeks, 6 months and 12 months (25).

Kasch et al. revisit results from a previously published cohort study that investigated motor and sensory dysfunction after whiplash injury—employing a unique study design using patients with ankle injuries as a control group. These authors performed a comprehensive assessment of pain, disability, nociceptive sensory processing and motor function across a 12-month period post-injury. Those patients that had reduced work capacity one year after injury had early reports of higher pain and demonstrated early sensory (cold pressor test, pressure pain) and motor (maximal voluntary contraction) dysfunction compared with whiplash patients that recovered and ankle-injured controls. These data suggest an interplay between patient symptoms, nociceptive sensitisation and motor control, that is associated with long term clinical outcome, which reflects the complexity of mechanisms underlying poor outcome after a whiplash injury.

Cervical sensorimotor control, and its association with patient clinical presentation, has been explored in relation to various types of neck pain. Sensorimotor deficits are arguably most relevant for WAD, considering its associated symptoms such as dizziness and unsteadiness. One of the most commonly used cervical sensorimotor control tests is the cervical movement sense test, in which a participant or patient with a laser pointer attached to the head, follows a target line as accurately as possible with controlled head movements. The study by Röijezon et al. investigates the discriminative validity and reliability of the cervical movement sense test, assessing the level of proprioceptive disturbance in people with WAD, idiopathic neck pain (INP), and healthy controls. Movement acuity was found to be a valuable outcome measure to assess disturbed cervical movement sense in both WAD and INP, with few differences in outcomes between the two neck pain groups. Yet, it was concluded that altered movement control strategies may exist between types of neck pain. Future investigation of such differential strategies can be used to inform management approaches tailored to WAD.

Alongside investigations of pain mechanisms, assessments, and the effectiveness of various treatments, there has been an increased emphasis on the clinical translation and implementation of evidence-based and high-value care approaches. As the clinical management of WAD remains to be challenging and development of physiotherapeutic care continues, a focus on quality improvement is warranted. Quality indicators can be used to standardise the assessment of such improvements, however, evidence lacks concerning their assessment, management, and patient-related outcome measures. Oostendorp et al. provide a synthesis of recent literature on quantitative measures of quality improvement in physiotherapy care. Included were more than 800 patients with WAD and a set of quality indicators used over a 16-year period. The authors conclude that physiotherapeutic care of WAD has substantially improved, and provide detailed suggestions for continued quality assurance.

The combined articles in this Research Topic illustrate the variety of work being undertaken to improve the understanding and clinical management of WAD. Building on the themes presented across these articles, further investigation is required to better understand the association between biopsychosocial mechanisms and the clinical presentation of patients with WAD and the symptoms they experience. Recent investigations into the (causal) relationship between genetic factors, biological mechanisms, and chronic pain presentations (26, 27) are a promising avenue to further explore the importance of these factors for the clinical management of WAD. Management approaches with demonstrable effects should be explored in large scale clinical trials. Preventing the transition from acute pain to a chronic condition, or the management of chronic pain itself, requires interventions with proven, clinically meaningful effects. Current evidence suggests that physical exercise, education, and psychological approaches should be considered as mainstay management for chronic WAD (9), however a more personalized approach for the prescription of these treatments may be needed. The implementation of evidence-based practice and utilization of clinical guidelines (28) can, and arguably should, be supported by reliable measures of quality improvement. A potential mechanism as posed by Oostendorp and colleagues is pursuing international consensus on quality monitoring and improvement, an initiative to be considered for improving targeted care for WAD.

## Author contributions

All authors contributed to manuscript preparation and decision to submit for publication. All authors approved the submitted version.

## References

[1] Hincapié CA Cassidy JD Côté P Carroll LJ Guzmán J. Whiplash injury is more than neck pain: a population-based study of pain localization after traffic injury. J Occup Environ Med. (2010) 52(4):434–40. 10.1097/JOM.0b013e3181bb806d 20357684

[2] Motor Accident Insurance Commission. *Annual CTP scheme insights: 2020–21*. (2021). Available from: https://maic.qld.gov.au/publications/annual-ctp-scheme-insights-2020-21/

[3] Holm LW Carroll LJ Cassidy JD Hogg-Johnson S Côté P Guzman J The burden and determinants of neck pain in whiplash-associated disorders after traffic collisions: results of the Bone and Joint Decade 2000–2010 Task Force on Neck Pain and its Associated Disorders. Spine. (2008) 33(4S):S52–9. 10.1097/BRS.0b013e3181643ece 18204401

[4] Sterling M Hendrikz J Kenardy J. Compensation claim lodgement and health outcome developmental trajectories following whiplash injury: a prospective study. PAIN. (2010) 150:22–8. 10.1016/j.pain.2010.02.013 20307934

[5] Griffin A Leaver A Moloney N. General exercise does not improve long-term pain and disability in individuals with whiplash-associated disorders: a systematic review. J Orthop Sports Phys Ther. (2017) 47(7):472–80. 10.2519/jospt.2017.7081 28622749

[6] Gross A Forget M St George K Fraser MM Graham N Perry L Patient education for neck pain. Cochrane Database Syst Rev. (2012) 3:CD005106. 10.1002/14651858.CD005106.pub4 PMC12042649 22419306

[7] Southerst D Nordin MC Côté P Shearer HM Varatharajan S Yu H Is exercise effective for the management of neck pain and associated disorders or whiplash-associated disorders? A systematic review by the Ontario Protocol for Traffic Injury Management (OPTIMa) Collaboration. Spine J. (2016) 16(12):1503–23. 10.1016/j.spinee.2014.02.014 24534390

[8] Teasell RW McClure JA Walton D Pretty J Salter K Meyer M A research synthesis of therapeutic interventions for whiplash-associated disorder (WAD): part 2—interventions for acute WAD. Pain Res Manag. (2010) 15(5):295–304. 10.1155/2010/640164 21038008 PMC2975532

[9] Sterling M de Zoete RMJ Coppieters I Farrell SF. Best evidence rehabilitation for chronic pain part 4: neck pain. J Clin Med. (2019) 8(8):1219. 10.3390/jcm8081219 31443149 PMC6723111

[10] Farrell SF Smith AD Hancock MJ Webb AL Sterling M. Cervical spine findings on MRI in people with neck pain compared with pain free controls: a systematic review and meta-analysis. JMRI. (2019) 49(6):1638–54. 10.1002/jmri.26567 30614121

[11] Bogduk N. On cervical zygapophyseal joint pain after whiplash. Spine. (2011) 36(25S):S194–9. 10.1097/BRS.0b013e3182387f1d 22020612

[12] Farrell SF Sterling M Irving-Rodgers H Schmid AB. Small fibre pathology in chronic whiplash-associated disorder: a cross-sectional study. Eur J Pain. (2020) 24(6):1045–57. 10.1002/ejp.1549 32096260

[13] White L Smith AD Farrell SF. Associations between resting heart rate, resting blood pressure, psychological variables and pain processing in chronic whiplash-associated disorder: a cross-sectional study. Pain Med. (2022) 19:pnac075. 10.1093/pm/pnac075 PMC9629357 35587744

[14] Van Oosterwijck J Nijs J Meeus M Paul L. Evidence for central sensitization in chronic whiplash: a systematic literature review. Eur J Pain. (2013) 17:299–312. 10.1002/j.1532-2149.2012.00193.x 23008191

[15] Stone AM Vicenzino B Lim EC Sterling M. Measures of central hyperexcitability in chronic whiplash associated disorder—a systematic review and meta-analysis. Man Ther. (2013) 18:111–7. 10.1016/j.math.2012.07.009 22947552

[16] Bontinck J Lenoir D Cagnie B Murillo C Timmers I Cnockaert E Temporal changes in pain processing after whiplash injury, based on Quantitative Sensory Testing: a systematic review. Eur J Pain. (2021) 26(1):227–45. 10.1002/ejp.1858 34464486

[17] Coppieters I Cagnie B Nijs J van Oosterwijck J Danneels L De Pauw R Effects of stress and relaxation on central pain modulation in chronic whiplash and fibromyalgia patients compared to healthy controls. Pain Physician. (2016) 19:119–30. 27008285

[18] Coppieters I De Pauw R Caeyenberghs K Danneels L Kregel J Pattyn A Decreased regional grey matter volume in women with chronic whiplash-associated disorders: relationships with cognitive deficits and disturbed pain processing. Pain Physician. (2017) 20:E1025–51. 10.36076/ppj/2017.7.E1025 29149149

[19] DePauw R Coppieters I Meeus M Caeyenberghs K Danneels L Cagnie B. Is traumatic and non-traumatic neck pain associated with brain alterations? – A systematic review. Pain Physician. (2017) 20:245–60. 28535548

[20] Sterling M Elliott JM Cabot PJ. The course of serum inflammatory biomarkers following whiplash injury and their relationship to sensory and muscle measures: a longitudinal cohort study. PLoS One. (2013) 8(10):e77903. 10.1371/journal.pone.0077903 24147095 PMC3798600

[21] Farrell SF de Zoete RMJ Cabot PJ Sterling M. Systemic inflammatory markers in neck pain: a systematic review with meta-analysis. Eur J Pain. (2020) 24(9):1666–86. 10.1002/ejp.1630 32621397

[22] Campbell L Smith A McGregor L Sterling M. Psychological factors and the development of chronic whiplash–associated disorder(s): a systematic review. Clin J Pain. (2018) 34(8):755–68. 10.1097/AJP.0000000000000597 29470185

[23] Sterling M Jull G Vicenzino B Kenardy J Darnell R. Physical and psychological factors predict outcome following whiplash injury. PAIN. (2005) 114(1–2):141–8. 10.1016/j.pain.2004.12.005 15733639

[24] Sterling M Jull G Kenardy J. Physical and psychological factors maintain long term predictive capacity following whiplash injury. Pain. (2006) 122(1–2):102–8. 10.1016/j.pain.2006.01.014 16527397

[25] Sterling M Smeets R Keijzers G Warren J Kenardy J Sterling M Physiotherapist-delivered stress inoculation training integrated with exercise versus physiotherapy exercise alone for acute whiplash-associated disorder (StressModex): a randomised controlled trial of a combined psychological/physical intervention. Brit J Sport Med. (2019) 53(19):1240–7. 10.1136/bjsports-2018-100139 30661011

[26] Farrell SF Campos AI Kho PF de Zoete RMJ Sterling M Rentería ME Genetic basis to structural grey matter associations with chronic pain. Brain. (2021) 144(12):3611–22. 10.1093/brain/awab334 34907416

[27] Farrell SF Kho PF Lundberg M Campos AI Rentería ME de Zoete RMJ A shared genetic signature for common chronic pain conditions and its impact on biopsychosocial traits. J Pain. (2022) 14:S1526-5900(22)00431-X. 10.1016/j.jpain.2022.10.005 36252619

[28] Zadro J O’Keeffe M Maher C. Do physical therapists follow evidence-based guidelines when managing musculoskeletal conditions? Systematic review. BMJ Open. (2019) 9(10):e032329. 10.1136/bmjopen-2019-032329 31591090 PMC6797428

